# A pill for the partner via the chlamydia patient? Results from a mixed method study among sexual health care providers in the Netherlands

**DOI:** 10.1186/s12879-018-3139-0

**Published:** 2018-05-29

**Authors:** Anita C. Nanhoe, Maartje Visser, Jurriaan J. Omlo, Anita J. C. M. Watzeels, Ingrid V. van den Broek, Hannelore M. Götz

**Affiliations:** 1 Center for Research and Business Intelligence, Rotterdam, The Netherlands; 2 Centre for Infectious Disease Control, National Institute for Public Health and the Environment, Bilthoven, The Netherlands; 3 Department of Infectious Disease Control, Municipal Public Health Service Rotterdam-Rijnmond, Rotterdam, The Netherlands; 4 Department of Public Health, Erasmus MC—University Medical Center Rotterdam, Rotterdam, The Netherlands

**Keywords:** Chlamydia trachomatis, Patient initiated partner therapy, Partner notification, Expedited partner therapy, Sexually Transmitted Infections, Health Services Research

## Abstract

**Background:**

Chlamydia prevalence in the Netherlands remains high despite targeted efforts. Effective Partner Notification (PN) and Partner Treatment (PT) can interrupt transmission and prevent re-infections. Patient Initiated Partner Treatment (PIPT) may strengthen chlamydia control. This study explores the current practice of PN and PT, and benefits of, and barriers and facilitators for PIPT among professionals in sexual health care in the Netherlands.

**Methods:**

A qualitative study was performed among GPs, GP-assistants (GPAs), physicians and nurses working at Sexual Health Clinics (SHC) and key-informants on ethnical diversity using topic lists in focus groups (*N* = 40) and semi-structured questionnaires in individual interviews (*N* = 9). Topics included current practices regarding PN and PT, attitude regarding PIPT, and perceived barriers and facilitators for PIPT. Interviews were taped, transcribed verbatim, and coded using ATLAS.ti. A quantitative online questionnaire on the same topics was sent to all physicians and nurses employed at Dutch SHC (complete response rate 26% (84/321)).

**Results:**

The qualitative study showed that all professionals support the need for more attention to PN, and that they saw advantages in PIPT. Mentioned barriers included unwilling PN-behaviour, Dutch legislation, several medical considerations and inadequate skills of GPs. Also, concerns about limited knowledge of cultural sensitivity around PN and PT were raised. Mentioned facilitators of PIPT were reliable home based test-kits, phone-contact between professionals and notified partners, more consultation time for GPs or GPAs and additional training. The online questionnaire showed that SHC employees agreed that partners should be treated as soon as possible, but also that they were reluctant towards PIPT without counselling and testing.

**Conclusions:**

Professionals saw advantages in PIPT, but they also identified barriers hampering the potential introduction of PIPT. Improving PN and counselling skills with specific focus on cultural sensitivity is needed. PIPT could be considered for specific partners. PIPT in combination with home based testing and using e-healthcare should be further explored and developed.

## Background

Chlamydia re-infections are common and increase risk of complications and adverse reproductive outcomes [1, 2]. About one third of chlamydia re-infections in heterosexuals are caused by untreated partners [2–4], stressing the importance of Partner Notification (PN) and Partner Therapy (PT), in STI control [5, 6].

Expedited Partner Therapy (EPT) allows health care providers to provide STI-patients with a prescription or antibiotics for their sex partner(s) without an intervening medical evaluation of the partner(s) [7]. This can be done by handing out medication to the partner by the index-case, (Patient Delivered Partner Therapy (PDPT)) [8, 9], or by providing an accelerated prescription for the partner with medical history gained through a telephone-hotline or at a pharmacy (Accelerated Partner Therapy (APT)) [10, 11]. EPT is efficacious in reducing re-infections in index patients and increasing the number of partners treated [8, 12]. In the USA EPT is permissible in 38 states and potentially allowable in 11 states [13]. Still, uptake of EPT was around 50% in studies [14]. In the UK APT is seen as a viable option, but did not achieve high uptake in practice [10, 11, 15].

In the Netherlands EPT as practiced in the US is currently not allowed by law. Dutch guidelines advise PN for chlamydia for all partners in the last 6 months, and to treat current and most recent ex-partners presumptively, without awaiting test-results [16, 17]. However, as chlamydia prevalence remains high in the Netherlands despite targeted PN efforts, the use of EPT could be of substantial benefit.

In 2016, the project investigating options for Patient Initiated Contact treatment for Chlamydia in the Netherlands (PICC-UP) started, to investigate the potential of EPT and/or APT as an effective method for improving PT in heterosexual patients. In this study we define ‘Patient Initiated Partner Treatment’ (PIPT), which can include both EPT or APT. One aim was to gain insight into the current practice of PN and PT and into opinions regarding benefits of, and barriers and facilitators for PIPT among professionals in sexual health care in the Netherlands. Furthermore, as chlamydia is more common in specific migrant groups [18] we explored views about PN and beliefs, norms and acceptability of PIPT among these groups in key-informants from various cultural backgrounds.

Sexual health care in the Netherlands is mostly carried out through the general practitioners (GP), who do 2/3 of all STI consultations. Additionally, Sexual Health Clinics (SHC) provide free of charge care for high-risk groups. In this investigation, we use a qualitative study among health staff in GP-practices and SHC, and among key-informants with various ethnic/migratory origins and a quantitative survey among physicians and nurses in SHC to describe current practice as well as opinions and beliefs regarding PN, PT and PIPT in Dutch sexual health care.

## Methods

### Qualitative study

#### Recruitment

We performed focus group discussions with SHC professionals (physicians and nurses) from various clinics and geographical areas who were recruited via their professional association. GPs were recruited for focus group discussions during a national meeting of all GPs specialized in sexual health care and additional individual interviews were held with GPs and practice assistants (GPAs) from both urban and more remote areas. Furthermore, group- and individual interviews were held with eight key informants with various cultural backgrounds, including experienced peer educators in a sexual health programme from the Municipal Public Health Service Rotterdam-Rijnmond. Table 1 shows information on gender and migratory background of the participants.

**Table 1:** Study population of qualitative study

Profession	Focus group	Individual	Total	Sex (F/M)	Migratory background
GPs	21	4	25	11/14	Dutch
GPAs	0	3	3	3/0	2 Dutch, 1 Turkish
SHC physicians	8	0	8	4/4	Dutch
SHC nurses	4	1	5	5/0	Dutch
Key-informants on ethnic diversity#	7	1	8	4/4	Diverse ^a^
TOTAL	40	9	49	27/ 22	

^a^The key-informants were Turkish (2 M), Surinamese (Creole and Hindustani) (1 M, 2 F), Antillean (1 F), Cape Verdean (1 M) and Dutch (1 F)

#### Data collection and analyses

Current practice of PN and PT was assessed with examples from practice. A semi structured topic list was developed based on literature, covering attitude towards PIPT, potential barriers and facilitators, and questions about possible options for PIPT in the Dutch situation. The predefined topics were discussed in each focus group (1–1.5 h) under the guidance of one facilitator and one observer. The individual interviews (30 min) were conducted either face to face or by phone, in accordance with the availability of the professionals. Data collection took place in the period January – June 2016. All interviews and focus group discussions were taped with consensus of the professionals and transcribed verbatim. The transcriptions were coded and analysed by AN and JO, with ATLAS.Ti 7 software for qualitative research. We opted for an inductive thematic analysis starting with a close line-by-line reading of the transcript, and developing from there, a conceptual or coding scheme. For triangulation purposes, the data were analysed individually by AN and JO and both themes and findings were discussed with AW and HG. The coded material, the analyses and the results were discussed by all authors.

### Quantitative survey

An online, anonymous, questionnaire was sent in September 2016 to all 73 physicians and 248 nurses employed at the 24 Dutch SHC. A reminder could not be sent. The questionnaire was self-developed and based on results of the qualitative study, and included seven-point Likert-type scale questions on current practice of PN and PT as well as on opinions and attitudes towards PIPT. The questionnaire was piloted with several research colleagues. Descriptive analyses were performed. Answers to the questions were summarised into disagreement (1–3 points) and agreement (5–7 points) versus neutral (4 points). Percentages were calculated excluding missing values.

## Results

### Qualitative study

The qualitative study included 25 GPs, 3 GPAs, 8 SHC-physicians, 5 SHC-nurses, and 8 key-informants (see Table 1 for characteristics of participants).

#### Current practice regarding PN

All professionals indicated to inform their patients about the diagnosis, treatment and preventive advice including a period to abstain from sexual contact. The SHC professionals applied multiple strategies to stimulate PN: giving factual information, counselling, motivational interviewing, and addressing the need of responsibility for the health of patient and partners in an understanding and sympathetic manner. These strategies were also named by the 3 GPAs interviewed and by some, but not all, GPs.

*‘Motivational interviewing can help you get closer to a solution step by step. It often works better, because every step comes from the patient. We as professionals can give all kind of advice, but if a patient doesn’t get it, then it just won’t work’ (GPA, female, Dutch).*

Professionals address the responsibility of patients for their own and their partner’s health. While some GPs use an authoritarian way, others prefer another style:

*One GP explains: ‘I do not think it will work to tell patients that they must notify partners. I rather tell them that they may have infected partners and explain about complications to motivate them to take their responsibility’(GP, female, Dutch).*

SHC and GP-practice professionals all have access to the PN-website “www.partnerwaarschuwing.nl” to support PN [19]. SHC health workers use this site regularly, as well as some GPAs.

#### Cultural differences in PN

The interviewed key-informants on ethnical diversity stressed that there should be more awareness among health professionals regarding differences between patients of various migratory origins regarding PN. They explained that the social pressure among people of Turkish, Moroccan and Hindustani origin is high when the honour of the family is at stake. Sometimes patients may therefore prevail their honour over their health.*‘Turkish people don’t just marry a woman, they marry a family. They’ll consider everything. They would tell it [red: diagnosis of STI] one to one, but the family circle makes it difficult’ (Key informant, male, Turkish).*

The Cape Verdean, Antillean and Surinamese key-informants indicated to experience a lower barrier for PN, because they are more open about sexuality than Moroccans or Turks. Within all mentioned migrant groups, men talk easier about STIs than women. There are in-group differences between generations and between people with and without a migration background. Key-informants mentioned that second generation young Turks and Cape Verdeans often disapprove of adultery. First generation Turks exhibit avoidance behaviour to prevent a divorce. Both attitudes are a barrier for PN. For younger Cape Verdeans extramarital sexual contacts are taboo, while multiple sexual contacts are viewed as experimental behaviour in youngsters not yet married, which makes PN less difficult for the latter group. Despite differences between and within groups, all key-informants had the impression that patients with a migratory origin tend to avoid PN.

#### Current practice regarding PT for chlamydia

At the SHC, notified partners have high priority in the triage and are tested following test-guidelines. Those notified by a current partner are offered treatment at first consultation, before test-results are known, usually as observed therapy and in some clinics through a prescription. Notified partners who consult their GP are often – though not always – tested before treatment. The majority of GP-practices limit their PT to the regular partner, and only when partner and index are registered at the same practice, because then the medical history of the partner is known. As a GP said:

‘*As a doctor we are responsible when we prescribe antibiotics and something goes wrong. That is a reason why we don’t prescribe for patients who are not registered with us’ (GP, female, Dutch).*

Despite several objections, GPs sometimes prescribe extra antibiotics for uninsured partners or when the patient finds PN too difficult. For example, if the patient fears domestic violence or - within certain migrant groups - honour related violence:

‘*If their safety is endangered, I give them an extra dose. The patients then put pills in the yogurt or tell the spouse that it’s for a sore throat’ (GP, female, Dutch).* This anecdotal quote as well as information from key informants showed that in extreme cases professionals search for ways to prevent re-infection of their patients outside any guideline.

#### General attitude regarding PIPT

All professionals saw several advantages in the use of PIPT. They thought that PIPT would treat more chlamydia infections in partners and consequently decrease the number of re-infections. Another advantage would be that partners who fail to get tested would be treated. Also, costs for testing may be avoided. However, the positive attitude of most professionals was quickly followed by a list of barriers and questions concerning how PIPT could be arranged in a legal and responsible way.

#### Professionals’ perceived barriers to using PIPT

##### Patients’ PN behaviour

The most mentioned barrier for PIPT concerned patients’ PN-behaviour. Execution of PN usually depends completely on the patient. Professionals mentioned that shame, fear, feelings of guilt and lack of motivation often stand in the way of PN. Patients with casual sex contacts often lack motivation to notify their partners or do not have contact information. GPs and GPAs mentioned a lack of ways to apply current partner immediately to enforce PN. Professionals can only notify a partner after the patient provides contact details of a partner and asks them to notify the partner, and only notified partners can be given PIPT.

##### Professionals’ knowledge and competencies

Several GPs and GPAs noted that GPs might need more specific training to talk about PN and PT, as some GPs feel uncomfortable discussing sexual issues with patients. One GP noted that younger GPs manage to discuss sexual issues easier:

*‘Young colleagues feel more at ease in talking about sexual issues. The young GP found a number of chlamydia infections by asking those patients uninhibitedly about their sex life and advising them a test. The older GP had no idea that these patients might be infected.’ (GP, male, Dutch).*

Some professionals thought that some GPs may not have enough knowledge on STIs and therefore do not provide their patients with crucial advice.

*‘I give a lot of training to GPs. When I ask: ‘How long should someone withhold from sex?’That’s when they ask me: ‘Can’t they have sex right away?’ (SHC physician female, Dutch).*

Especially the GPAs mentioned the need for training of GPs in counselling and motivational interviewing, in giving the right information, addressing responsibility in a non-judgemental way, and referring patients to “partnerwaarschuwing.nl”.

The key-informants additionally suggested communication training concerning cultural sensitivity, as several ethnical groups experience a taboo on sexuality and STIs which may influence strategies for PN and PIPT.

##### Legal barriers

Another often mentioned barrier to PIPT was the legislation. Physicians are only allowed to hand out medication after personal contact with a patient and after checking contra-indications such as medicine use and allergies.

*One SHC physician mentioned: “I think we doctors are stricter in this, as we are responsible and liable for prescription of medication. We have the legal situation in mind. Nurses may be more pragmatic, but I feel strongly responsible and do think that we cannot just do something with medication”(SHC physician, female, Dutch).*

Physicians feared liability issues when giving (a prescription for) extra antibiotics for partners without knowing possible contra-indications or for someone not being their patient.

##### Medical barriers

Various medical safety concerns were mentioned by the professionals. First, physicians feared giving extra antibiotics for partners without knowing possible contra-indications:

*‘The risk something goes wrong, is not high, but still I wouldn’t want to take the risk, as it could have some consequences. I don’t see other ways than checking allergies and contra-indications for Azithromycin. Although I realize that* PIPT *will lower the threshold and a larger number of chlamydia cases will be treated’ (GP, female, Dutch).*

The question was also raised how the prescription would be written in the partners’ patient file, and how medication would be delivered. Furthermore, professionals noted that handing out extra antibiotics, without consultation, feels like incomplete care. Partners might not be infected with chlamydia at all, might have an anorectal chlamydia infection or have other STIs, requiring different treatment. Use of PIPT might in this case offer a false sense of security and lead to untreated infections:

*‘Some GPs hand out extra pills when there’s a high risk the patient won’t notify the partner. But it’s better to notify the partners, so that they have the possibility to choose for a proper test, as there could be other STIs’ (GPA, female, Dutch).*

Also mentioned was the importance of testing and counselling the partner to determine whether the partner was infected, or might have infected other sex partners. Several professionals also wondered whether patients and partners will change their sexual behaviour when antibiotics are handed out without counselling. Especially professionals from SHC preferred counselling on STI and sexual behaviour before handing out antibiotics:

*‘It’s a missed opportunity if the partner receives the medication through the patient and learns nothing from it’(SHC physician, female, Dutch).*

Last, the need for careful use of antibiotics and avoiding overtreatment and potential risk of antibiotic resistance was mentioned by several physicians.

#### Facilitators for PIPT

The professionals also posed several ideas that could facilitate the use of PIPT. GPs indicated to need more than their usual consultation time for STI-patients. As a GPA says: *‘When you have two minutes consultation time left, you tend to work towards a conclusion. So the limited time makes it difficult’(GPA, female, Turkish).* Referring STI-patients to the GPAs for additional counselling and discussion of PN and PIPT could also solve this problem. Another facilitator for providing PIPT would be to enable (phone)-contact with the partner to inquire about allergies and medication before handing out medication for the partner to the index-patient. The possibility of a hot line for partners to discuss their questions with a health care professional was also perceived as helpful.

Furthermore, most professionals regarded an STI test prior to PT essential. Facilitating STI testing of the partner would therefore also facilitate the use of PIPT among professionals. Even when partners after taking samples at home would wait for the test results before taking the medication, the period between treating the patient and partner would be less than when they have to make an appointment. Home-based testing could be used together with PIPT where the partner is given a home based sampling test-kit via the index and sends the sample to the SHC to receive their results. This would also provide partners a feeling of anonymity.

*‘People don’t have to go anywhere and they can decide for themselves where and when they do the test’ (SHC physician, male, Dutch).*

Last, professionals indicated that if PIPT were to be implemented, they would like to receive proper training in its use and information on how and for whom it should be available.

### Quantitative survey

The overall response rate of the quantitative survey was 36% (115/321). The 115 respondents were from 23 out of 24 SHC. 32% (80/248) of the nursing staff responded and 48% (35/73) of the physicians. Hardly any differences were seen between answers of physicians and nurses therefore the overall responses are reported. Only 84 out of the 115 respondents completed the whole survey, resulting in a complete response rate of 26% (84/321). Partially completed questionnaires were included in the analyses.

#### Current practice of PN and PT

PN is discussed with the index-patient during the treatment consultation. SHC employ different methods of informing patients about their STI test results. On clinic level, 26% (6/23) send a text-message to the patient about the result; 30% (7/23) phone the patient, 13% (3/23) do both text-message and phone-call and 30% (7/23) provide patients a code to login and view their results on the clinic website. 57% (17/30) of professionals of clinics who phone the patient about results directly offer a double consultation for the patient and partner. This percentage was 29% (11/38) and 7% (2/31) in clinics working with text-message and login codes respectively.

Assessment of the current practice of PT showed that almost all professionals adhere to the current guidelines. When partners are presenting at the clinic for consultation, 97% (97/100) of professionals treat the current partner immediately, and also take an STI test. Only 3% (3/91) of professionals stated to use PIPT for the current partner (but not ex-partners) when knowing that the partner does not have contra-indications.

#### Considerations for potential implementation of PIPT

**Fig. 1:**
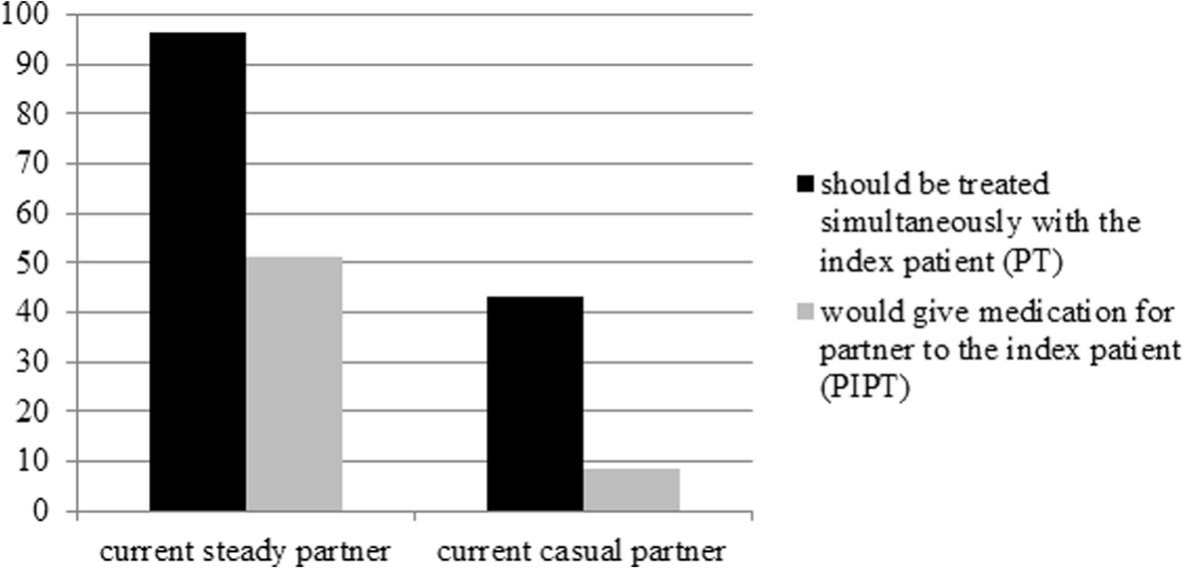
Differences in attitude towards (patient initiated) partner therapy for steady and casual partners among Dutch SHC employees

When asked about the importance of partner treatment, 97% (83/86) of respondents agreed that current steady partners should get treatment immediately (43% (37/86) for the casual partners). A much smaller proportion would consider providing PIPT: 51% (43/84) for a steady and 8% (7/84) for a casual partner (Fig. 1).

**Fig. 2:**
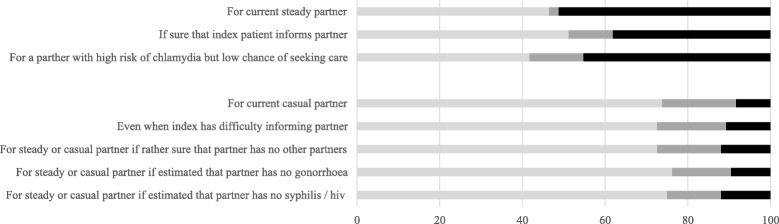
Attitude towards potential implementation of EPT for different types of partners and situations among Dutch SHC employees

We asked the professionals about their willingness to provide PIPT in different situations. Professionals were most likely to consider PIPT if the partner had a high risk of chlamydia and a low chance of presenting for a consultation (45%; 38/84), and when they knew that the index-patient would inform the current partner (38%; 32/84). Only 11% (9/84) would hand out medication in cases when the index patient finds it difficult to inform their partner. The percentage of professionals considering PIPT for both current and casual partners was rather low, around 10–12%, even when the professional would know that the partner had no other partners, or could estimate that the partner would not have gonorrhoea of syphilis or HIV infection (Fig. 2).

#### Conditions for PIPT

Checking for allergies and contra-indications of the partner before prescribing/handing out azithromycin was considered necessary by 97% (83/86) of the professionals. When asked via which route this should be done, 62% (53/86) preferred a personal consultation, 19% (16/86) thought this could be done by telephone, 13% (11/86) thought this would be possible by internet and 7% (6/86) agreed this could be done at the pharmacy.

**Fig. 3:**
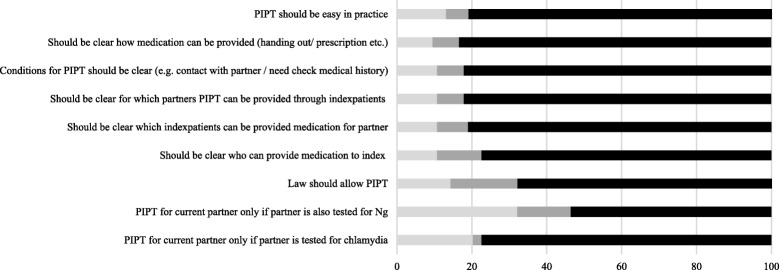
Attitudes towards conditions for potential implementation of EPT among Dutch SHC employees

When asked about potential risks of PIPT, 79% (68/86) thought it would be risky to provide PIPT as it is unknown what the index patient will do with it. 49% (42/86) agreed that PIPT would encourage unnecessary antibiotic prescription, increasing the risk of antibiotic resistance development, while 31% (27/86) disagreed. Differences in attitude towards conditions for PIPT among the STI clinic employees are shown in Fig. 3. Testing for chlamydia in case of providing PIPT was considered conditional by 77% of professionals, but only half (54% (45/84)) would also require testing for gonorrhoea. 68% (57/84) agreed that PIPT can only be given if the law would allow this. Clarity about who could provide medication, to which index-patients, for which partners and how the partner’s medical history can be checked was considered essential by around 80% of 84 professionals. Last, the majority 81% (68) agreed that PIPT should be easy in practice.

## Discussion

### Main findings

PN and selection of partners eligible for presumptive treatment are prerequisites for PIPT. We found that the majority of SHC professionals follow the guidelines for treating current regular partners presenting at their clinic. Professional attitudes and competencies as well as partner characteristics and cultural backgrounds of patients explain barriers in PN and PT. In general, both GP-practice and SHC professionals were on one hand convinced that PIPT would have many advantages such as preventing re-infections, but on the other hand were critical and reluctant towards PIPT without counselling and testing.

### Strengths and limitations

A drawback of the quantitative study is the relatively low response rate of the internet questionnaires. This could have led to biased results. However, many of the findings from the qualitative interviews were confirmed by the questionnaire, which contributes to the reliability of our results. The combination of the qualitative interviews followed by an internet survey among SHC staff is thus a strength of this study. Furthermore, the interviews included multiple kinds of sexual health care professionals from large cities and rural areas all over the country. A limitation is that the focus groups in this study are homogenous groups of Dutch professionals. All participants that were interviewed have special interest in STIs in their work and may therefore be particularly favourable for innovative ideas, and not be representative for all professionals. However, this limitation is also a strength of this study as these professionals have expertise and insight in the policy, legislation and treatment of STIs and may be the innovators in case of implementation of PIPT [20].

We interviewed experienced peer educators in the field of sexual health care from various cultural backgrounds. A limitation is that these key-informants on ethnical and cultural sensitivity are expressing both their professional and personal views, which may be biased. However, cultural sensitivity is important when choosing a strategy for PN, PT and PIPT, and therefore this theme requires and deserves more research.

### Comparison to other countries and studies

#### Practice of PN

PN is a condition for PIPT. It is therefore not surprising that perceived barriers concern PN and counselling in the first place. Professionals combine several strategies for discussing PN with their patients, which they perceived as supportive and effective. Especially motivational interviewing helps patients to formulate their own needs and possible solutions. There is room for improvement with regard to counselling competencies of GPs [21]. A difference between professionals at GP practices and SHC is that the latter are used to address sexual health in a neutral way and use motivational interviewing [22–24], while some GPs seem to show uneasiness in discussing sexual health and use authoritative and judging manners in communication with patients.

Although it was previously found that (intimate) partner violence was not a major concern for PN and PIPT, this may deserve attention in specific groups [25, 26]. This is exemplified by the anecdotal quote of a GP providing a prescription also for the partner who may be prone to violence, realising that the patient may administer the pills secretly.

#### PT and PIPT

A prerequisite for PIPT is selection of partners eligible for presumptive PT. In both the qualitative and the quantitative study we found that the guidelines for treating current regular partners are followed once partners do present in care, however current casual partners are less often treated. Also many partners present with a delay after treatment of the index patient. SHC inform patients about their test results by phone, text-message or with a code to check the results on the internet. It appeared that the more personal contact a SHC has when informing the index-patient, the earlier PN, testing and treatment is discussed. Here the personal approach in smaller clinics is advantageous as compared to creating efficiency in large clinics [27].

In both studies we found that professionals agreed that PT should be improved and that PIPT would be supportive in prevention of chlamydia re-infections in patients. When assessing barriers for PIPT we found three main themes: legal and medical arguments and issues concerning assessment of risk profiles of partners.

#### Legal arguments

Similar to previous studies, physicians were concerned about obeying the law, and feared liability problems, for example when treating persons who are not their own patients [28, 29].

In the Netherlands it is required prior to prescription of medication that the physician has contact with the partner, assesses the medical history (allergies and contra-indications) and explains the reason for prescription. Not all physicians may be fully aware of the legal situation. PIPT is prescribed by GPs in 4–6% of patients, mostly for partners who are patients in the same practice [30]. Whether the partner is informed about the reason for prescription is unclear as is whether GPs are fully aware of legal requirements. It has been reported before that professionals who are given instructions about the legal impossibilities are reluctant in the use of PIPT, while those not exactly knowing what is allowed had used PIPT increasingly [31].

Respondents stressed that it should be very clear what is allowed with respect to PIPT before they would use it, which is in correspondence with findings in other studies [28, 29, 31, 32].

#### Medical arguments

Professionals considered a personal consultation with partners imperative, for evaluation of medical history and for preventive messages. Despite agreement that PT should be provided to current partners, the percentage of professionals who would use PIPT was much lower. This reluctant attitude can be explained by the responsibility they felt about the safety of partners and the concern whether medication would reach the right person and whether the adequate medication would be provided, for example because anal infections should be treated with Doxycycline instead of Azithromycin. Similar medical concerns have been reported before [31–33]. Another concern was potential side effects of Azithromycin, although respondents acknowledged that in fact side effects would be scarce. No serious adverse effects have been reported from Azithromycin associated with EPT trials or subsequent surveillance in the US, leading to the suggestion that jurisdictions should endorse EPT [34]. These medical arguments could be counteracted by APT, where contact with a partner is a condition met.

Although PIPT might have beneficial effects, there were concerns about the missed opportunity to screen a high-risk population for STI other than chlamydia [35, 36]. A part of the PICC-UP study was the assessment of STI in SHC clients who were notified for chlamydia. Treating them without gonorrhoea testing would miss 10 % of gonorrhoea in this high risk population [37]. Partner testing was perceived as highly important for using PIPT. Combining testing and treating would reduce the delay in PT. Providing a home based test kit for the partner may even facilitate PN as the index-patient has something to offer, and would prevent unnecessary use of antibiotics when a partner would wait for the result [38]. If the partner has an STI, PN can be extended to the sex-network of the partner.

#### Assessment of risk profiles of partners

Only one in ten SHC professionals would provide PIPT when estimating that the partner has no other partners or STIs. This may be due to the difficulties in estimating risk profiles of partners. Patients do not necessarily know what their partners do. It is therefore understandable that professionals regard personal contact with the partner essential. Having telephone contact with partners has been found as a facilitator for PIPT before [6] but contacting a partner during the treatment consultation may pose logistic problems. Options like a hotline as in APT may be a possibility [11]. Only few professionals were open for assessing the medical history by internet. Provided that this is legally allowed in SHC, an online service combined with traditional service as described recently may be the way forward [39].

## Conclusions and recommendations

Physicians and nurses from SHC and GP practice staff found PT for chlamydia important, but their attitude towards PIPT was reluctant. PIPT could be considered for steady partners or for partners who may not present for testing, but contact with the partner is deemed essential. Partner testing was perceived as an important condition for using PIPT. Thus a form of APT could be the way to go. APT in combination with home based testing and using e-healthcare should be further explored and developed. Before implementing APT, improving PN and counselling skills with specific focus on cultural sensitivity is needed.

## Abbreviations

APT: Accelerated partner therapy
EPT: Expedited partner therapy
GP: General practitioner
GPA: General practitioner assistant
PDPT: Patient delivered partner therapy
PIPT: Patient initiated partner therapy
PN: Partner notification
PT: Partner therapy
SHC: Sexual Health Clinic

## References

[1] Haggerty CL, Gottlieb SL, Taylor BD, Low N, Xu F, Ness RB (2010). Risk of sequelae after chlamydia trachomatis genital infection in women. J Infect Dis.

[2] Hosenfeld C, Workowski K, Bouman S, Zaidi A, Dyson J, Mosure D, Bolan G, Bauer H (2009). Repeat infection with chlamydia and gonorrhea among females: a systematic review of the literature. Sex Transm Dis.

[3] Gotz HM, van den Broek IV, Hoebe CJ, Brouwers EE, Pars LL, Fennema JS, Koekenbier RH, van Ravesteijn S, Opdecoul EL, van Bergen J (2013). High yield of reinfections by home-based automatic rescreening of chlamydia positives in a large-scale register-based screening programme and determinants of repeat infections. Sex Transm Infect.

[4] Gotz HM, Wolfers ME, Luijendijk A, van den Broek IV (2013). Retesting for genital chlamydia trachomatis among visitors of a sexually transmitted infections clinic: randomized intervention trial of home- versus clinic-based recall. BMC Infect Dis.

[5] Cowan FM, French R, Johnson AM (1996). The role and effectiveness of partner notification in STD control: a review. Genitourin Med.

[6] Ferreira A, Young T, Mathews C, Zunza M, Low N (2013). Strategies for partner notification for sexually transmitted infections, including HIV. Cochrane Database Sys Rev.

[7] Expedited partner therapy in the management of sexually transmitted diseases https://www.cdc.gov/std/treatment/eptfinalreport2006.pdf. Accessed 24 Apr 2018.

[8] Golden M, Whittington W, Handsfield H, Hughes J, Stamm W, Hogben M, Clark A, Malinski C, Helmers J, Thomas K (2005). Effect of expedited treatment of sex partners on recurrent or persistent gonorrhea or chlamydial infection. N Engl J Med.

[9] Golden MR, Kerani RP, Stenger M, Hughes JP, Aubin M, Malinski C, Holmes KK (2015). Uptake and population-level impact of expedited partner therapy (EPT) on chlamydia trachomatis and Neisseria gonorrhoeae: the Washington state community-level randomized trial of EPT. PLoS Med.

[10] Estcourt CS, Sutcliffe LJ (2008). Accelerated partner therapy. Int J STD AIDS.

[11] Estcourt CS, Sutcliffe LJ, Copas A, Mercer CH, Roberts TE, Jackson LJ, Symonds M, Tickle L, Muniina P, Rait G (2015). Developing and testing accelerated partner therapy for partner notification for people with genital chlamydia trachomatis diagnosed in primary care: a pilot randomised controlled trial. Sex Transm Infect.

[12] Althaus CL, Turner KM, Mercer CH, Auguste P, Roberts TE, Bell G, Herzog SA, Cassell JA, Edmunds WJ, White PJ (2014). Effectiveness and cost-effectiveness of traditional and new partner notification technologies for curable sexually transmitted infections: observational study, systematic reviews and mathematical modelling. Health Technol Assess.

[13] Legal Status of EPT - Summary Totals. https://www.cdc.gov/std/ept/legal/totals.htm. Accessed 14 May 2018.

[14] Schillinger JA, Gorwitz R, Rietmeijer C, Golden MR (2016). The expedited partner therapy continuum: a conceptual framework to guide programmatic efforts to increase partner treatment. Sex Transm Dis.

[15] Dombrowski JC, Golden MR (2012). Accelerated partner therapy: a promising new partner treatment option. Sex Transm Infect.

[16] Van Bergen J, Dekker J, Boeke A, Kronenberg E, Van der Spruit R, Burgers J, Bouma M, Verlee E (2013). Dutch GP guideline on STI consultations [guidelines in Dutch: NHG-Standaard Het soa-consult (Eerste herziening)]. Huisarts en Wetenschap.

[17] Draaiboek Partnermanagement http://www.rivm.nl/dsresource?objectid=c65d5b96-abda-4157-8737-e52a11c68ff6. Accessed 24 Apr 2018.

[18] Visser M, Van Aar F, Van Oeffelen AAM, van den Broek IVF, Op de Coul ELM, Hofstraat SHI, Heijne JC, den Daas C, Hoenderboom BM, van Wees DA, VAF VM, AAM v O, den Broek IVF v, de Coul ELM O, SHI H, JCM H, den Daas C, Hoenderboom BM, Van Wees DA, Basten M, Woestenberg PJ, Götz HM, Van Sighem AI, de Hoon S, BHB VB (2017). Sexually transmitted infections including HIV, in the Netherlands in 2016. RIVM report.

[19] Gotz HM, van Rooijen MS, Vriens P, Op de Coul E, Hamers M, Heijman T, van den Heuvel F, Koekenbier R, van Leeuwen AP, Voeten HA (2014). Initial evaluation of use of an online partner notification tool for STI, called 'suggest a test': a cross sectional pilot study. Sex Transm Infect.

[20] Grol R, Wensing M. Implementatie, effectieve verbetering in de patiëntenzorg. Amsterdam: Reed Business BV; 2011.

[21] Temkin E, Klassen AC, Mmari K, Gillespie DG (2011). A qualitative study of patients' use of expedited partner therapy. Sex Transm Dis.

[22] Lundahl B, Moleni T, Burke BL, Butters R, Tollefson D, Butler C, Rollnick S (2013). Motivational interviewing in medical care settings: a systematic review and meta-analysis of randomized controlled trials. Patient Educ Couns.

[23] Op de Coul EL, Spijker R, van Aar F, van Weert Y, de Bruin M (2013). With whom did you have sex? Evaluation of a partner notification training for STI professionals using motivational interviewing. Patient Educ Couns.

[24] Theunissen KA, Schipper P, Hoebe CJ, Crutzen R, Kok G, Dukers-Muijrers NH (2014). Barriers to and facilitators of partner notification for chlamydia trachomatis among health care professionals. BMC Health Serv Res.

[25] Rosenfeld EA, Marx J, Terry MA, Stall R, Pallatino C, Borrero S, Miller E. Intimate partner violence, partner notification, and expedited partner therapy: a qualitative study. Int J STD AIDS. 2015; 10.1177/0956462415591938 26088259

[26] Vaidya S, Johnson K, Rogers M, Nash D, Schillinger JA (2014). Predictors of index patient acceptance of expedited partner therapy for chlamydia trachomatis infection and reasons for refusal, sexually transmitted disease clinics, New York City, 2011 to 2012. Sex Transm Dis.

[27] Ling SB, Richardson DB, Mettenbrink CJ, Westergaard BC, Sapp-Jones TD, Crane LA, Nyquist AC, McFarlane M, Kachur R, Rietmeijer CA (2010). Evaluating a web-based test results system at an urban STI clinic. Sex Transm Dis.

[28] Pavlin NL, Parker RM, Piggin AK, Hopkins CA, Temple-Smith MJ, Fairley CK, Tomnay JE, Bowden FJ, Russell DB, Hocking JS (2010). Better than nothing? Patient-delivered partner therapy and partner notification for chlamydia: the views of Australian general practitioners. BMC Infect Dis.

[29] Shivasankar S, Challenor R (2008). Patient-delivered partner therapy in the UK: what do the professionals think?. Int J STD AIDS.

[30] van den Broek IVF, Donker GA, van Benthem BH, Van Bergen JEAM, Götz HM. Partner notification and partner treatment for chlamydia: attitude and practice of general practitioners in the Netherlands; a landscape analysis. BMC Fam Pract. 2017;18(1):103. 10.1186/s12875-017-0676-3 PMC5738758 29262799

[31] Rosenfeld EA, Marx J, Terry MA, Stall R, Pallatino C, Miller E (2015). Healthcare providers' perspectives on expedited partner therapy for chlamydia: a qualitative study. Sex Transm Infect.

[32] Rosenfeld EA, Marx J, Terry MA, Stall R, Flatt J, Borrero S, Miller E. Perspectives on expedited partner therapy for chlamydia: a survey of health care providers. International journal of STD & AIDS. 2016;27(13):1180–6. 10.1177/0956462415610689 26446138

[33] Cameron S, Glasier A, Muir A, Scott G, Johnstone A, Quarrell H, Oroz C, McIntyre M, Miranda D, Todd G (2010). Expedited partner therapy for chlamydia trachomatis at the community pharmacy. BJOG.

[34] Huffam SE, Brown KM, Chen MY, Fairley CK (2013). Legislate for patient-delivered partner therapy for chlamydia. Med J Aust.

[35] McNulty A, Teh MF, Freedman E (2008). Patient delivered partner therapy for chlamydial infection--what would be missed?. Sex Transm Dis.

[36] Stekler J, Bachmann L, Brotman RM, Erbelding EJ, Lloyd LV, Rietmeijer CA, Handsfield HH, Holmes KK, Golden MR (2005). Concurrent sexually transmitted infections (STIs) in sex partners of patients with selected STIs: implications for patient-delivered partner therapy. Clin Infect Dis.

[37] Gotz HM, van Aar F, Van Benthem BH: Concurrent STIs in sex partners notified for chlamydia: implications for patient initiated partner therapy in: IUSTI: 2016*;* IUSTI Budapest; 2016.

[38] Markos A (2008). Patient-delivered partner medication: the antagonism of clinical standards and good medical practice. Int J STD AIDS.

[39] Estcourt CS, Gibbs J, Sutcliffe LJ, Gkatzidou V, Tickle L, Hone K, et al. The eSexual Health Clinic system for management, prevention, and control of sexually transmitted infections: exploratory studies in people testing for Chlamydia trachomatis. Lancet Public Health. 2017;2(4):e182–e90. Epub 2017/12/19. 10.1016/S2468-2667(17)30034-8 29253450

